# Book Review: The Big Five in SLA

**DOI:** 10.3389/fpsyg.2021.710365

**Published:** 2021-06-25

**Authors:** Rui Teng, Honggang Liu

**Affiliations:** Northeast Normal University, Jilin, China

**Keywords:** The Big Five, SLA, psychology, personality, trait

The Big Five model, the major psychological model that describes human personality, has increasingly been the focus of research on second language acquisition (SLA). Although the significance of the Big Five traits is self-evident, little research has been done on this topic in SLA in comparison to other individual differences such as motivation. Therefore, *The Big Five in SLA*, by Professor Ewa Piechurska-Kuciel, is a timely book. It provides researchers in SLA with new information about how the Big Five model has been explored in recent years, and it offers future directions for research in this area.

The book is composed of four chapters. Chapter 1 introduces the definitions of personality and illustrates analytical approaches to personality from psychoanalytic, learning, and humanistic perspectives. It also addresses type theory and trait theory, the latter of which is the theoretical basis for the Big Five model. Chapter 2 reviews the Big Five model, the Five-Factor Theory (FFT) and trait hierarchy. Much of this chapter is devoted to the theoretical analysis of each trait: neuroticism, agreeableness, conscientiousness, extraversion, and openness to experience. Piechurska-Kuciel also explores the consequences of the five traits from the perspective of three categories: socioaffective, cognitive and academic, as well as behavioural. In Chapter 3, after a general review of the interdisciplinary uniqueness of SLA, Piechurska-Kuciel progresses through an examination of individual differences, personality and, finally, personality traits in SLA. Furthermore, she expounds upon the theoretical claims of future studies related to each of the five personality traits by reviewing the latest empirical studies. In the final chapter, based on existing studies on the role of each personality trait in foreign language learning, Piechurska-Kuciel identifies solutions to the inconsistencies seen in previous research results and she recommends future research directions, such as the relationship between extraversion and language learning emotion. She also explores the personality characteristics of successful foreign language learners at three levels, and she summarises some approaches and pedagogical interventions to enhance the strengths and avoid the weaknesses of each trait. For instance, agreeable students are skilled in creating a harmonious classroom atmosphere, but their exceedingly self-satisfied behaviour might be detrimental to learner autonomy. Therefore, tasks that require agreeable students to practise the language with classmates and then fulfil a subsequent assignment independently can beneficial.

This book is valuable to read for the following reasons. First, the Big Five model has been applied in various fields, one of which is SLA. However, the slow progress of studying the five personality traits in SLA demonstrates the inadequacy of the interface between the Big Five model and SLA. Most previous researchers have employed a single psychological perspective, but under the increasingly apparent interdisciplinary trend of SLA (Gass et al., 2020), it is necessary to draw from other disciplines to facilitate research studies on the Big Five in SLA. The book under review sets a great example in this respect, as it adopts a wide range of social, emotional, cognitive, academic, and behavioural perspectives to explore the five personality traits in SLA. This attempt transcends the limitations of the dominant psychological perspective; therefore, it enriches research on the Big Five in SLA. For example, an extensive amount of research on neuroticism has been conducted using a psychometric approach to explore its psychological nature. This unidimensional view has restricted the further examination of neuroticism, which is necessary since human beings are situated in complex social-cultural contexts. A psychological trait, such as neuroticism, may be associated with other aspects if we take a socioaffective approach. In Chapter 3, Piechurska-Kuciel discusses neuroticism from this perspective and provides new explanations for this trait in SLA. Neurotic learners, who often experience negative emotions, may find it stressful to contact teachers and classmates, and they may be anxious about communicating in a second language. This theme foregrounds the importance of the social context in SLA and its effects on personality traits. It offers evidence that particular personality traits (in this case, neuroticism) are socially-constructed, not static.

Second, Piechurska-Kuciel provides ideas for further theoretical and empirical research by reviewing her own studies and recent empirical studies by other researchers. The relationship between personality and SLA has been under-researched (Dewaele, 2012; Coker and Mihai, 2017). In the few studies that are available, the focus has been on the role of extroversion and openness to experience in SLA, but scant attention has been paid to neuroticism, agreeableness, and conscientiousness. Given this imbalance, Piechurska-Kuciel points out several under-explored topics, such as the links between preparedness in neurotic students and various forms of foreign language attainment that she addresses in Chapter 4. She also recommends topics to be investigated in the future; for instance, in Chapter 3 she suggests that researchers should delve more deeply into the role of conscientiousness in foreign language proficiency.

The Big Five model has made significant headway in psychology, but there is still the opportunity for further exploration in SLA. This book addresses this potential by expanding the research perspectives in this interdisciplinary era for SLA and extending the topics of research. This book will be a valuable reference for postgraduate students and researchers who take an interest in applying the Big Five model in SLA.

## Author Contributions

RT: draught and revision. HL: conceptulation, framework, supevision, and funding. Both authors contributed to the article and approved the submitted version.

## Conflict of Interest

The authors declare that the research was conducted in the absence of any commercial or financial relationships that could be construed as a potential conflict of interest.

## References

[B1] Coker C. Mihai F. (2017). Personality traits and second language acquisition: the influence of the Enneagram on adult ESOL students. TESOL J. 8, 432–449. 10.1002/tesj.281

[B2] Dewaele J.-M. (2012). “Personality: personality traits as independent and dependent variables”, in Psychology for Language Learning, eds Mercer S. Ryan S. Williams M. (New York, NY: Palgrave Macmillan) 42–57. 10.1057/9781137032829_4

[B3] Gass S. M. Behney J. Plonsky L. (Eds.). (2020). Second Language Acquisition: An Introductory Course. London: Routledge. 10.4324/9781315181752

